# Neutrophilia in the bronchoalveolar lavage fluid increases coughing during flexible fiberoptic bronchoscopy in a pediatric cohort

**DOI:** 10.3389/fped.2024.1347983

**Published:** 2024-07-08

**Authors:** Laura Danino, Florian Stehling, Maximilian Eckerland, Eser Orhan, Eva Tschiedel

**Affiliations:** ^1^ Department of Pediatric Pulmonology and Sleep Medicine, Children’s Hospital, University of Duisburg-Essen, Essen, Germany; ^2^ Department of Pediatric Research Network, Children's Hospital, University of Duisburg-Essen, Essen, Germany; ^3^ Department of Pediatric Intensive Care, Children’s Hospital, University of Duisburg-Essen, Essen, Germany

**Keywords:** bronchoscopy, bronchoalveolar lavage, neutrophils, cough, pediatrics, endoscopy

## Abstract

**Objective:**

This study is an addition to the already published prospective randomized double-blinded trial by Tschiedel et al. that compared two different sedation regimes in fiberoptic flexible bronchoscopy in pediatric subjects. The objective of the presented study is to analyze the correlation between the neutrophil percentage of the bronchoalveolar lavage fluid (BALF) and coughing episodes during bronchoscopy.

**Methods:**

Fifty subjects, aged 1–17 years, received flexible fiberoptic bronchoscopy under deep sedation. The BALF of 39 subjects was analyzed with reference to cytology and microbiology.

**Results:**

The percentage of neutrophils from the total cell count ranged from 0% to 95.3% (median 2.7). Nineteen patients (49%) had a percentage of ≥3.0%. Pearson's correlation showed a high correlation (*r *= 0.529, *p* = 0.001) between the coughing episodes per minute and the neutrophil percentage in the BALF. Analysis of variance showed a significant difference in neutrophil percentage between the indication groups (*p *= 0.013). The *t*-test (*p* = 0.019) showed a significant difference between the neutrophil percentage for patients with a probable airway infection under immunosuppression (median 2.9) and patients with cystic fibrosis (median 49.6). The linear regression analysis showed a significantly stronger impact of the neutrophil percentage on coughing frequency than the sedation regime (*β_neutrophils_* = 0.526 with *p* = 0.001 vs. *β_sedation_* = 0.165 with *p* = 0.251).

**Conclusion:**

When bronchoscopy is to be performed on a pediatric patient with suspected bacterial or viral infection, and therefore neutrophilic airway inflammation, coughing is to be expected.

## 1 Introduction

In pediatric flexible fiberoptic bronchoscopy, deep sedation of the patient is essential for a successful examination (1). The quality of the bronchoscopy and the number of complications during the procedure highly depend on the use of sedation. However, one major drawback that occurs during the performance of the procedure is in the form of disturbance caused by a coughing patient (2, 3). In children especially, the risk of inducing cough is high, since the ratio of pediatric airways to the used bronchoscopes is smaller than that of adults (4).

We previously published a prospective randomized double-blinded trial comparing the quality of two sedation regimes (with and without an opioid) with regard to the frequency of coughing episodes during bronchoscopy (5). It was shown that a sedation regime with propofol and remifentanil was superior to a regime with propofol alone in terms of cough frequency. In the presented study, the collected data were retrospectively analyzed again and combined with the simultaneously obtained neutrophil percentage of the findings of bronchoalveolar lavage (BAL).

It is known that chronic cough in pediatric patients is associated with significant neutrophilia and viral and/or bacterial infection in the BAL fluid (BALF) (6–8). However, it has never been examined whether airway neutrophilia also has a negative effect on coughing during the performance of pediatric bronchoscopy.

## 2 Method

A detailed description of the prospective randomized double-blinded trial has been provided by Tschiedel et al. (1). In brief, 50 subjects aged 1–17 years were included (mean 6.8, standard deviation 5.6 years), and of these, 23 were female. All subjects received flexible fiberoptic bronchoscopy for diagnostic reasons and were randomized in two groups. In 39 subjects, a BAL was performed. In all subjects, the indication for the bronchoscopy and the BAL was determined by a pediatric pulmonologist (Table 1).

**Table 1:** Indications for bronchoscopy with BAL.

Indication for bronchoscopy	Number of subjects	Percentage of subjects
Evaluation of cough	9	23.1
Evaluation of possible respiratory infection	9	23.1
Evaluation of possible respiratory infection in cystic fibrosis	6	15.4
Evaluation for congenital malformation	5	12.8
Evaluation of possible respiratory infection under immunosuppression	4	10.2
Evaluation of possible chronic/immunologic lung disease	3	7.7
Evaluation of hemoptysis	2	5.1
Evaluation of difficult-to-treat asthma	1	2.6
Total	39	100

The subjects were categorized in groups by indication for the bronchoscopy and the BAL (Table 1). Retrospectively, we identified one patient who was diagnosed with cystic fibrosis, which was not known in the initial study. Neither the frequency nor the quality of cough prior to the performance of the bronchoscopy was documented. However, in nine subjects, persistent cough was the reason for performing the procedure.

All bronchoscopies were performed by a pediatric pulmonologist in accordance with ATS guidelines (9). The bronchoscopes used had an outer diameter ranging between 2.8 and 3.8 mm. Sedation was performed by pediatric intensivists in accordance with international guidelines (10).

The sedation regime was double-blinded (group PP: propofol and placebo; group PR: propofol and remifentanil). The groups were compared in terms of frequency of coughing during the bronchoscopy as a primary outcome parameter. One coughing episode was defined as an episode of continuous coughing without inspiration. In this study, the results of the BAL cytology and microbiology analyses were collected. The BAL data were retrospectively used to additionally analyze the correlation of the number of coughing episodes per minute and the percentage of neutrophils in the BALF. Since the Shapiro–Wilks test showed that all parameters were normally distributed (*p* < 0.001), we used Pearson's correlation. BAL cytologies from 39 of the 50 subjects were available and included in the analysis. A neutrophil percentage ≥3.0% was defined as pathological in accordance with the ERS task force on BAL in children (11). In 39 patients, the BALF was analyzed for bacteria and fungi, and in 36 patients, it was also analyzed for viruses.

To compare the neutrophil percentage between the indication groups for the procedure, we performed a one-way analysis of variance (ANOVA).

In addition, we compared the coughing frequency in the indication groups (Table 1). Subjects with expected high levels of neutrophilia in the BALF (cystic fibrosis) were compared with immunosuppressed subjects who were likely to have less neutrophilic airway inflammation (12–14). In the immunosuppressed cohort, three of the four subjects had neutropenia when bronchoscopy was performed. We then employed Student’s t-test to compare the groups.

We used Pearson’s correlation to examine the correlation between the duration of sedation with the coughing frequency.

To further address the effect of the neutrophil percentage and the effect of the sedation regime on the coughing episodes per minute, we performed a linear regression analysis.

A statistical analysis was performed using SPSS statistical software (version 27, IBM SPSS Inc.). A value of *p* < 0.05 was considered statistically significant with a confidence interval of 95%.

Our amendment was approved by the local ethics committee (14-5883-BO).

## 3 Results

In 39 subjects, the BALF were analyzed.

Table 2 shows the microbiological findings. In 51.3% of the BALF, no bacterial specimen was found, and in 52.8%, no virus was found. Most of those bacteria and viruses were common respiratory pathogens associated with the underlying diseases.

**Table 2:** Microbiological findings in the BALF.

Microorganism	Number of subjects	Percentage of BAL analyzed
Bacterial (*n* = 39)		
*Haemophilus influenzae*	7	17.9
*Staphylococcus aureus*	4	10.2
*Streptococcus pneumoniae*	3	7.7
*Moraxella catarrhalis*	2	5.1
*Streptococcus pyogenes*	1	2.6
*Streptococcus* group G	1	2.6
*Pseudomonas aeruginosa*	1	2.6
*Mycobacterium tuberculosis*	1	2.6
*Escherichia coli*	1	2.6
*Achromobacter xylosoxidans*	1	2.6
No findings	20	51.3
Viral (*n* = 36)		
Rhinovirus	12	33.3
Adenovirus	3	8.3
Coronavirus (non-SARS, non-MERS)	3	8.3
Parainfluenza virus	2	5.5
Bocavirus	2	5.5
Enterovirus	1	2.8
No findings	19	52.8
Fungi (*n* = 39)		
*Aspergillus fumigatus*	2	5.1
No findings	37	94.9

SARS, severe acute respiratory syndrome; MERS, Middle East respiratory syndrome.

**Figure 1:**
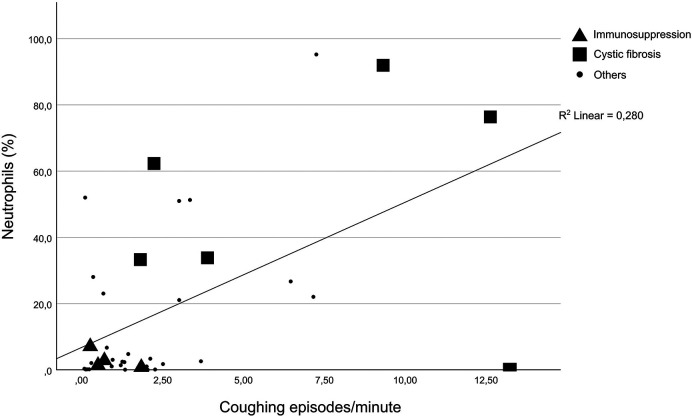
Correlation between neutrophils in the BALF and coughing episodes per minute.

The cytology results are provided in Table 3. The percentage of neutrophils from the total cell count ranged from 0% to 95.3% (median 2.7). Nineteen subjects (49%) had a percentage of ≥3.0%. Pearson’s correlation showed a positive correlation between the coughing episodes per minute and the percentage of neutrophils measured in the BALF (*r *= 0.529, *p* = 0.001, Figure 1).

**Table 3:** BALF cytology cell percentages.

Cells	Range (in % of total cell count)	Median (in % of total cell count)
Macrophages	0–100	64
Lymphocytes	0–87.3	14.3
Granulocytes	0–96.5	3.3
Neutrophils	0–95.3	2.7
Eosinophils	0–14.3	0.3
Mast cells	0–3.7	0
Plasma cells	0	0

In addition, ANOVA showed a significant difference in neutrophil percentage between the indication groups (*p *= 0.013).

The t-test (*p* = 0.019) showed a significant difference between the neutrophil percentages for patients with a probable airway infection under immunosuppression (median 2.925) and patients with cystic fibrosis (median 49.6). In Figure 1, patients with cystic fibrosis and those under immunosuppression are highlighted.

The duration of sedation did not influence the coughing frequency (Pearson's correlation was 0.141 with *p* = 0.391).

The linear regression analysis indicated that the neutrophil percentage had a stronger impact on coughing episodes per minute than the sedation regime (*β_neutrophils_* = 0.526 with *p* = 0.001 was higher than *β_sedation_* = 0.165 with *p* = 0.251).

## 4 Discussion

Neutrophils in the BALF are an indicator of bacterial and/or viral inflammation (6, 7, 14). In pediatric asthma, as well as primary ciliary dyskinesia, coughing correlates with neutrophils in the sputum (15, 16).

The presented study is the first to identify a risk factor for coughing during flexible bronchoscopy in pediatric patients. It shows a significant correlation between the percentage of neutrophils found in the BALF and the frequency of coughing during a flexible bronchoscopy performed under deep sedation in a pediatric cohort. The higher the percentage of neutrophils in the BALF, the higher the risk of coughing during the procedure. Inflamed airway mucosa is more susceptible to irritation than healthy mucosa during bronchoscopy.

In addition, despite the small number of subjects, we were able to show that patients with cystic fibrosis have a higher probability for a high neutrophil percentage and are therefore more likely to cough during the performance of bronchoscopy, which can complicate the procedure. However, patients under immunosuppression are likely to have a small neutrophil percentage and will probably cough less.

Furthermore, we were able to show that neutrophil percentage has a higher impact on coughing than the sedation regimes used in this study.

In conclusion, when bronchoscopy is to be performed in a pediatric patient with suspected bacterial or viral infection and therefore neutrophilic airway inflammation, coughing is to be expected during the procedure, especially in patients with cystic fibrosis. In this context, we suggest that the examiner should be well-experienced to minimize risk and to optimize the quality of the bronchoscopy procedure.

A limitation of this study is the small-sized and heterogeneous cohort. In addition, in the initial study, two different sedation regimes were compared. One can speculate that with a different regime, coughing may be suppressed more effectively, although it has already been proved that remifentanil has a potent antitussive effect. However, a different regime may be associated with other adverse events such as desaturations and hypoxemia.

Further studies to identify risk factors for pediatric flexible bronchoscopy as well as sedation regimes that successfully suppress disturbances such as cough are warranted.

## Data Availability

The raw data supporting the conclusions of this article will be made available by the authors without undue reservation.
